# Neural Tube Defects in Pregnancies Among Women With Diagnosed HIV Infection — 15 Jurisdictions, 2013–2017

**DOI:** 10.15585/mmwr.mm6901a1

**Published:** 2020-01-10

**Authors:** Jennita Reefhuis, Lauren F. FitzHarris, Kristen Mahle Gray, Steven Nesheim, Sarah C. Tinker, Jennifer Isenburg, Benjamin T. Laffoon, Joseph Lowry, Karalee Poschman, Janet D. Cragan, Fay K. Stephens, Jane E. Fornoff, Cheryl A. Ward, Tri Tran, Ashley E. Hoover, Eirini Nestoridi, Laura Kersanske, Monika Piccardi, Morgan Boyer, Mary M. Knapp, Abdel R. Ibrahim, Marilyn L. Browne, Bridget J. Anderson, Dipal Shah, Nina E. Forestieri, Jason Maxwell, Kimberlea W. Hauser, Godwin U. Obiri, Rachel Blumenfeld, Dana Higgins, Carla P. Espinet, Bernardita López, Katherine Zielke, Latoya P. Jackson, Charles Shumate, Kacey Russell, Margaret A. Lampe

**Affiliations:** ^1^Division of Birth Defects and Infant Disorders, National Center on Birth Defects and Developmental Disabilities, CDC; ^2^Division of HIV/AIDS Prevention, National Center for HIV/AIDS, Viral Hepatitis, STD, and TB Prevention, CDC; ^3^ICF, Atlanta, Georgia; ^4^Florida Department of Health; ^5^Metropolitan Atlanta Congenital Defects Program, Atlanta, Georgia; ^6^HIV/AIDS Epidemiology Section, Georgia Department of Public Health; ^7^Adverse Pregnancy Outcomes Reporting System, Illinois Department of Public Health; ^8^HIV Surveillance Program, Illinois Department of Public Health; ^9^Louisiana Birth Defects Monitoring Network, Louisiana Office of Public Health; ^10^STD/HIV Program, Louisiana Office of Public Health; ^11^Massachusetts Center for Birth Defects Research and Prevention, Massachusetts Department of Public Health; ^12^Bureau of Infectious Disease and Laboratory Sciences, Massachusetts Department of Public Health; ^13^Office for Genetics and People with Special Health Care Needs, Maryland Department of Health; ^14^Center for HIV Surveillance, Epidemiology and Evaluation, Maryland Department of Health; ^15^Division of Family Health Services, Early Identification and Monitoring, New Jersey Department of Health; ^16^Division of HIV, STD and TB Services, New Jersey Department of Health; ^17^Congenital Malformations Registry, New York State Department of Health; ^18^Bureau of HIV/AIDS Epidemiology, New York State Department of Health; ^19^HIV Epidemiology and Field Services Program, New York City Department of Health and Mental Hygiene, New York; ^20^Birth Defects Monitoring Program, North Carolina Department of Health and Human Services; ^21^Division of Public Health, Communicable Disease Branch, North Carolina Department of Health and Human Services; ^22^Bureau of Epidemiology, Pennsylvania Department of Health; ^23^HIV Surveillance & Epidemiology, Pennsylvania Department of Health; ^24^Division of Maternal, Child and Family Health, Philadelphia Department of Public Health; ^25^AIDS Activities Coordinating Office, Philadelphia Department of Public Health, Pennsylvania; ^26^Birth Defects Surveillance and Prevention System, Puerto Rico Department of Health; ^27^HIV/AIDS Surveillance Program, Puerto Rico Department of Health; ^28^Bureau of Health Improvement and Equity, South Carolina Department of Health and Environmental Control; ^29^Bureau of Communicable Disease and Prevention, South Carolina Department of Health and Environmental Control; ^30^Birth Defects Epidemiology and Surveillance Branch, Texas Department of State Health Services; ^31^TB/HIV/STD Epidemiology and Surveillance Branch, Texas Department of State Health Services.

In May 2018, a study of birth defects in infants born to women with diagnosed human immunodeficiency virus (HIV) infection in Botswana reported an eightfold increased risk for neural tube defects (NTDs) among births with periconceptional exposure to antiretroviral therapy (ART) that included the integrase inhibitor dolutegravir (DTG) compared with other ART regimens (*1*). The World Health Organization* (WHO) and the U.S. Department of Health and Human Services^†^ (HHS) promptly issued interim guidance limiting the initiation of DTG during early pregnancy and in women of childbearing age with HIV who desire pregnancy or are sexually active and not using effective contraception. On the basis of additional data, WHO now recommends DTG as a preferred treatment option for all populations, including women of childbearing age and pregnant women. Similarly, the U.S. recommendations currently state that DTG is a preferred antiretroviral drug throughout pregnancy (with provider-patient counseling) and as an alternative antiretroviral drug in women who are trying to conceive.^§^ Since 1981 and 1994, CDC has supported separate surveillance programs for HIV/acquired immunodeficiency syndrome (AIDS) (*2*) and birth defects (*3*) in state health departments. These two surveillance programs can inform public health programs and policy, linkage to care, and research activities. Because birth defects surveillance programs do not collect HIV status, and HIV surveillance programs do not routinely collect data on occurrence of birth defects, the related data have not been used by CDC to characterize birth defects in births to women with HIV. Data from these two programs were linked to estimate overall prevalence of NTDs and prevalence of NTDs in HIV-exposed pregnancies during 2013–2017 for 15 participating jurisdictions. Prevalence of NTDs in pregnancies among women with diagnosed HIV infection was 7.0 per 10,000 live births, similar to that among the general population in these 15 jurisdictions, and the U.S. estimate based on data from 24 states. Successful linking of data from birth defects and HIV/AIDS surveillance programs for pregnancies among women with diagnosed HIV infection suggests that similar data linkages might be used to characterize possible associations between maternal diseases or maternal use of medications, such as integrase strand transfer inhibitors used to manage HIV, and pregnancy outcomes. Although no difference in NTD prevalence in HIV-exposed pregnancies was found, data on the use of integrase strand transfer inhibitors in pregnancy are needed to understand the safety and risks of these drugs during pregnancy.

In the United States, many aspects of adult HIV surveillance are standardized across all 50 states, the District of Columbia, and six territories, but surveillance for pregnancy outcomes among women with diagnosed HIV infection varies across jurisdictions (*2*). A comprehensive national surveillance approach for birth defects does not exist. Not all jurisdictions have birth defects surveillance programs, and among those that do, there is variability in surveillance methods and in the program’s authority to ascertain cases that end in a stillbirth or termination. Active birth defects surveillance programs send abstractors to hospitals and other data sources to identify pregnancies affected by birth defects; passive birth defects surveillance programs receive notifications from hospitals and health care practitioners about pregnancies affected by birth defects, and some passive surveillance programs use a hybrid method where notifications lead to abstractions for verifying reported cases (*4*).

CDC contacted the 20 jurisdictions with the highest numbers of women of reproductive age living with diagnosed HIV infection that also had birth defects surveillance programs with data available from 2013 to 2017. This period was chosen to ascertain birth defects during the 5 years after DTG was approved for use in the United States by the Food and Drug Administration in 2013. Certain jurisdictions were not able to obtain the required legal agreements between the different governmental departments responsible for each program to perform the data linkage or were otherwise not able to contribute to this effort.

After obtaining required agreements, the birth defects surveillance programs in 15 jurisdictions (including 11 states, Atlanta metropolitan area, New York City, Philadelphia, and Puerto Rico) identified pregnancies affected by NTDs (on the basis of *International Classification of Diseases, Ninth Revision, Clinical Modification* [ICD-9-CM] code range 740–742.0 and ICD-10-CM codes Q00.0–Q01.9, Q05.0–Q05.9, Q07.01, and Q07.03) for the period 2013 through 2017. U.S. jurisdictions have varying levels of authority to ascertain nonlive births. For this report, pregnancies include live births, stillbirths, and induced terminations. Identifying data for the mothers was matched to HIV surveillance records, using locally established linking algorithms, to ascertain whether any data related to the women with an NTD-affected pregnancy were also available in the HIV surveillance system. 

Total population prevalence estimates for NTDs were calculated by dividing the number of pregnancies affected by NTDs by the total number of live births during 2013–2017 in the reporting jurisdictions. Denominators for prevalence calculations of HIV-exposed births were the number of live births that occurred during 2013–2017 among women with diagnosed HIV infection. To establish these denominators, most jurisdictions matched HIV surveillance data to birth certificates; one state used data from their comprehensive newborn HIV screening program. Variability was assessed using 95% confidence intervals (CIs) calculated with the Poisson methods. Nonoverlapping confidence intervals were used as a measure of statistical difference to acknowledge the imprecision of the estimate on the basis of small numbers. SAS (version 9.4; SAS Institute) was used to conduct all analyses.

Participating jurisdictions^¶^ had surveillance information on 64,272 women aged 13–44 years with diagnosed HIV infection in 2015,** which represents approximately 70% of all women aged 13–44 years living with diagnosed HIV infection in the United States in 2015. Among 8,043,489 live births from these jurisdictions during 2013–2017, the prevalence of NTDs was 5.8 per 10,000 live births (Table). Data linkage between the two independent surveillance systems in each jurisdiction identified eight NTD cases, and there were 11,425 live births to women with diagnosed HIV infection during 2013–2017, for a prevalence of 7.0 per 10,000 HIV-exposed live births; this did not significantly differ from the general population prevalence, on the basis of the overlapping confidence intervals.

**TABLE:** Neural tube defect (NTD) prevalence among the general population of births and human immunodeficiency virus (HIV)–exposed births in 15 jurisdictions — United States, 2013–2017*

Type of BD surveillance	General population births			HIV-exposed births		
	Total no. of live births	NTDs	NTDs per 10,000 live births (95% CI^§^)	Total no. of live births	NTDs	NTDs per 10,000 live births (95% CI^§^)
**All jurisdictions^†^**	**8,043,489**	**4,656**	**5.8 (5.6–6.0)**	**11,425**	**8**	**7.0 (3.0–13.8)**
Active BD surveillance^¶^	3,850,065	2,685	7.0 (6.7–7.2)	4,697	3	6.4 (1.3–18.7)
Passive BD surveillance^¶^	4,193,424	1,971	4.7 (4.5–4.9)	6,728	5	7.4 (2.4–17.3)
Only ascertain BD in live births**	2,194,646	1,261	5.7 (5.4–6.1)	3,681	4	10.9 (3.0–27.8)
Ascertain BD in live births and nonlive births**	5,848,843	3,395	5.8 (5.6–6.0)	7,744	4	5.2 (1.4–13.2)

**Abbreviations**: BD = birth defects; CI = confidence interval.

* Florida provided data from 2013 to 2015.

^†^ Data from Philadelphia and New York City were included in the data for Pennsylvania and New York State, respectively; however, it is important to note that the majority of HIV-exposed pregnancies originated from the metropolitan jurisdictions. Data from the Metropolitan Atlanta Congenital Defects Program (MACDP) represents three counties in Georgia: DeKalb, Fulton, and Gwinnett; the other jurisdictions are statewide.

^§^ Calculated using exact Poisson methods because of the small number of cases.

^¶^ Jurisdictions with active BD surveillance: MACDP within Georgia, Louisiana, Massachusetts, North Carolina, Puerto Rico, South Carolina, and Texas; jurisdictions with passive BD surveillance: Florida, Illinois, Maryland, New Jersey, New York, and Pennsylvania.

** Jurisdictions that only ascertain BD in live births: Florida, Louisiana, New Jersey, and Pennsylvania; jurisdictions that ascertain BD in live births and nonlive births: MACDP within Georgia, Illinois, Maryland, Massachusetts, New York, North Carolina, Puerto Rico, South Carolina, and Texas.

For the general population in these 15 jurisdictions, the NTD prevalence was higher when the analysis was limited to active surveillance programs (7.0 per 10,000 live births), which have more complete data than do passive programs (4.7 per 10,000 live births) (Table). Among women with diagnosed HIV infection, the NTD prevalence estimates based on active and passive surveillance had overlapping confidence intervals, suggesting no difference on the basis of case ascertainment. Surveillance systems that are not able to ascertain birth defects among nonlive births will usually underascertain NTDs because pregnancies affected by NTDs often lead to nonlive births. However, for these 15 jurisdictions, the NTD prevalence estimates for the general population and NTD prevalence estimates for pregnancies of women with diagnosed HIV infection were considered similar among programs that did or did not include nonlive births because the respective confidence intervals were wide and overlapped.

## Discussion

For the first time, linked data from HIV and birth defects surveillance programs were used to estimate the prevalence of birth defects among pregnancies among women with diagnosed HIV infection. The prevalence of NTDs among pregnancies among women with diagnosed HIV infection in these 15 jurisdictions (7.0 per 10,000 live births) does not appear to differ from all births in these jurisdictions and from the estimate for the U.S. population based on 24 states (approximately 8 per 10,000 live births) (*5*,*6*). However, an association between ART and NTDs could not be assessed because information about maternal ART use is not collected routinely.

Additional pregnancies were followed up in Botswana, and two studies (*7*,*8*) have reported that risks of NTDs are lower than suggested by the initial study (*1*) (threefold versus eightfold, respectively). WHO now recommends DTG as a preferred treatment option for all populations, including women of childbearing age and pregnant women based on an evaluation of both risks and benefits.

The HHS Panel on Treatment of Pregnant Women with HIV Infection and Prevention of Perinatal Transmission now recommends DTG as a preferred antiretroviral drug throughout pregnancy and as an alternative antiretroviral drug in women who are trying to conceive, and also strongly recommends that use of DTG be accompanied by appropriate counseling to allow joint decision-making between patients and providers. CDC is exploring data on ART and birth defects that can be compiled in the United States. The Antiretroviral Pregnancy Registry (*9*) has provided some data to assess this association, but the addition of a U.S. population-based estimate, not dependent on volunteer participation, would be an important addition to the literature. CDC is currently working with partners to use the linked data in this report to ascertain specific ART use before or during early pregnancy and specific NTD phenotypes as well as other birth defects.

The findings in this report are subject to at least five limitations. First, the birth defects surveillance data might have been incomplete because surveillance methods varied by jurisdiction, nonlive birth outcomes were not available in all jurisdictions, and 2017 data might have been incomplete because of delays in abstraction. Second, linkage of persons’ data in two separate surveillance programs is never 100% complete because of differences in linking variables, such as names or birth dates, which could have resulted in underestimation of the total number of births and NTDs. Third, approximately one in nine women with HIV have not received a diagnosis and therefore are not monitored by HIV surveillance.^††^ Fourth, because of data limitations, it was not possible to adjust for confounders. Finally, CIs were used as a measure of variability, and nonoverlapping CIs were considered statistically different. This analytical approach is considered a conservative evaluation of significance differences and infrequently can lead to the conclusion that estimates are similar, even when point estimates do differ significantly.

Because data on pregnancy and ongoing antiretroviral medication use are not routinely collected in many state HIV surveillance programs, and HIV treatment options are evolving, continued efforts to collect information on pregnancies affected by maternal HIV infection are needed to understand the association between HIV treatment and birth defects and other pregnancy outcomes. Linkage of data from other surveillance programs might help to assess possible associations between maternal disease or maternal use of medications, and pregnancy outcomes.

**Summary**

**What is already known about this topic?**

In 2018, an association between periconceptional dolutegravir exposure and neural tube defects (NTD) was reported in Botswana. Data from U.S. birth defects and human immunodeficiency virus/acquired immunodeficiency syndrome (HIV/AIDS) surveillance programs had not previously been linked to assess NTD prevalence in births to women with HIV.

**What is added by this report?**

Linking of data from birth defects and HIV/AIDS surveillance programs in 15 jurisdictions was done for the first time. The NTD prevalence in HIV-exposed pregnancies during 2013–2017 was estimated to be 7.0 per 10,000 live births, similar to the prevalence in the general population in the 15 jurisdictions and the U.S. estimate.

**What are the implications for public health practice?**

Current U.S. recommendations state that dolutegravir is a preferred antiretroviral drug throughout pregnancy (with provider-patient counseling) and an alternative antiretroviral drug in women who are trying to conceive. Although no difference in NTD prevalence in HIV-exposed pregnancies was found, data on the use of integrase strand transfer inhibitors in pregnancy are needed to understand the safety and risks of these drugs during pregnancy.

## Footnotes

* https://www.who.int/medicines/publications/drugalerts/Statement_on_DTG_18May_2018final.pdf.

† https://aidsinfo.nih.gov/news/2094/statement-on-potential-safety-signal-in-infants-born-to-women-taking-dolutegravir-from-the-hhs-antiretroviral-guideline-panels; https://aidsinfo.nih.gov/news/2109/recommendations-regarding-the-use-of-dolutegravir-in-adults-and-adolescents-with-hiv-who-are-pregnant-or-of-child-bearing-potential.

§ https://www.who.int/news-room/detail/22-07-2019-who-recommends-dolutegravir-as-preferred-hiv-treatment-option-in-all-populations; https://aidsinfo.nih.gov/guidelines/html/3/perinatal/224/whats-new-in-the-guidelines.

¶ Florida, Georgia (metropolitan Atlanta), Illinois, Louisiana, Maryland, Massachusetts, New Jersey, New York City, New York State, North Carolina, Pennsylvania, Philadelphia, Puerto Rico, South Carolina, and Texas.

** https://www.cdc.gov/nchhstp/atlas/index.htm.

†† https://www.cdc.gov/hiv/group/gender/women/index.html.
